# Transformation of the output of spinal lamina I neurons after nerve injury and microglia stimulation underlying neuropathic pain

**DOI:** 10.1186/1744-8069-3-27

**Published:** 2007-09-27

**Authors:** A Florence Keller, Simon Beggs, Michael W Salter, Yves De Koninck

**Affiliations:** 1 Unité de Neurobiologie Cellulaire, Centre de Recherche Université Laval Robert-Giffard, Québec, QC G1J 2G3, Canada; 2 Hospital for Sick Children, University of Toronto, Toronto, ON, Canada

## Abstract

**Background:**

Disinhibition of neurons in the superficial spinal dorsal horn, via microglia – neuron signaling leading to disruption of chloride homeostasis, is a potential cellular substrate for neuropathic pain. But, a central unresolved question is whether this disinhibition can transform the activity and responses of spinal nociceptive output neurons to account for the symptoms of neuropathic pain.

**Results:**

Here we show that peripheral nerve injury, local spinal administration of ATP-stimulated microglia or pharmacological disruption of chloride transport change the phenotype of spinal lamina I output neurons, causing them to 1) increase the gain of nociceptive responsiveness, 2) relay innocuous mechanical input and 3) generate spontaneous bursts of activity. The changes in the electrophysiological phenotype of lamina I neurons may account for three principal components of neuropathic pain: hyperalgesia, mechanical allodynia and spontaneous pain, respectively.

**Conclusion:**

The transformation of discharge activity and sensory specificity provides an aberrant signal in a primarily nociceptive ascending pathway that may serve as a basis for the symptoms of neuropathic pain.

## Background

Neuropathic pain is a widespread and highly debilitating condition commonly resulting from injury to peripheral nerves or from a variety of causes including trauma, cancer, HIV-AIDS or diabetes [1]. Unlike inflammatory pain, for which many efficacious treatments exist, neuropathic pain is typically refractory to most current treatments and thus represents a major unmet medical need. Key symptoms of neuropathic pain are hyperalgesia, allodynia and spontaneous pain. Hyperalgesia involves enhanced pain perception to noxious stimuli; allodynia designates pain experienced in response to an innocuous stimulus and spontaneous pain refers to recurring pain, not necessarily related to an identifiable peripheral stimulus. Of these symptoms, tactile allodynia (e.g. pain induced by gentle mechanical stimulation of the skin) and spontaneous pain are the most prevalent and debilitating [2]. Several cellular substrates for these symptoms have been proposed [3], including suppression of the inhibition mediated by GABA_A_- and glycine receptors in the dorsal horn of the spinal cord [4-6]. We have discovered that following peripheral nerve injury such disinhibition of spinal dorsal horn neurons occurs by a collapse of the anion gradient in lamina I neurons [7] via a novel microglia-neuron signalling pathway [8], leading to weakening GABA_A_- and glycine-mediated inhibitory synaptic transmission [9,10].

Lamina I is one of the main nociceptive output pathways from the spinal cord to the brain and, in contrast to lamina V neurons, lamina I neurons do not receive direct input from low-threshold Aδ and Aβ afferents [11]; rather they normally encode and transmit essentially noxious or thermal information [12-15]. However, tactile allodynia requires that innocuous inputs elicit a nociceptive percept or response. Because lamina I output neurons do not normally respond to innocuous mechanical input [14], it is unclear how disinhibition of these neurons might cause innocuous input to trigger a noxious sensation at the supraspinal level. Similarly, spontaneous pain requires ongoing or episodic activity in nociceptive pathways, and lamina I nociceptive output neurons have little or no spontaneous activity [14]. In the present study, we investigated the possibility that peripheral nerve injury and microglia-driven disinhibition in the dorsal horn transform lamina I output neurons from silent and strictly responsive to noxious stimuli to allow these neurons to display spontaneous activity and be driven additionally by innocuous peripheral stimulation.

## Results

### Characterization of the neuronal population

**Figure 1:**
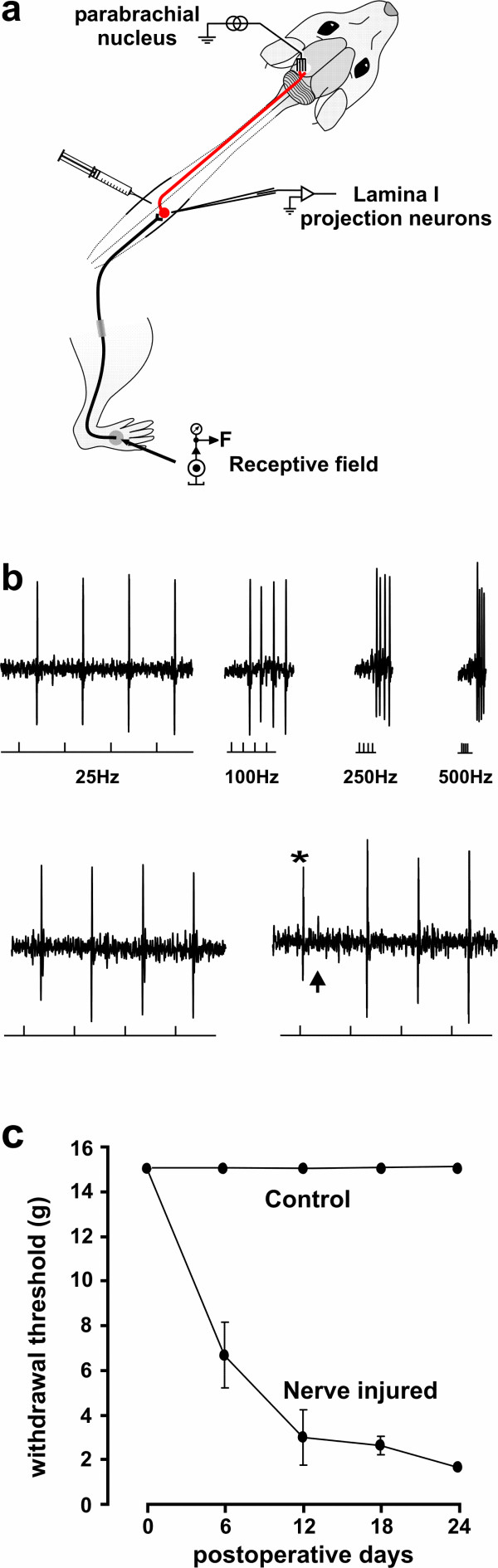
**Experimental paradigm**. **a**. Schematic representation of the experimental setting to record from single antidromically identified lamina I projection neurons. Cells were recorded from control animals and animals that received a chronic constriction injury of the sciatic nerve. **b**. Confirmation of recording from lamina I projection neuron. *Top*: extracellular single unit recordings from a lamina I neuron showing one-for-one following of a train of antidromic stimuli (lower traces mark the stimulus; 1 mA, 200 μs duration; up to 500 Hz) delivered from the electrode positioned in the lateral parabrachial nucleus. Conduction distance was 100 mm. *bottom*: collision of the first of 4 antidromic action potentials (25 Hz) with an orthodromic action potential (*) occurring within the critical interval. The arrow points to the position where the first antidromic action potential would have occurred in absence of the orthodromic action potential (as in the trace on the left). **c**. Graph showing results of nociceptive reflexes to mechanical stimuli of the rats included in the current study. Peripheral nerve injury (N = 12) caused a significant reduction of the withdrawal threshold to mechanical stimulation of the hind paw. Nerve injured animals were taken between 16 and 24 days post-injury. Animals were anesthetized with pentobarbital or ketamine/xylazine and single unit extracellular recording was performed from lamina I projection neurons identified by antidromic stimulation from the parabrachial nucleus.

We used extracellular single unit recording *in vivo *in rats to study the responses to natural peripheral stimuli of spinal lamina I projection neurons [7] (Fig. 1a). Each neuron studied was confirmed to project to the parabrachial nucleus (Fig. 1b), which is richly innervated by terminals of lamina I neurons in the rat [12]. The lamina I spino-parabrachial pathway is known to encode nociceptive sensory information [16,17] and play a critical role in integrating cardiovascular, autonomic and emotional responses to pain [18]. Thus, the action potential discharge of spino-parabrachial lamina I neurons studied here represents a primarily nociceptive output of the spinal dorsal horn.

We recorded from a total of 50 confirmed lamina I spino-parabrachial projection neurons in 38 control rats and in 12 rats with a chronic construction injury to the sciatic nerve. Nociceptive withdrawal threshold was measured for each rat included in the study (Fig. 1c). The average depth of recording, location of the receptive field, threshold for antidromic stimulation and conduction velocity of ascending axon of lamina I neurons were not different between naïve and nerve injured rats (Table 1), indicating that the types or location of neurons recorded were comparable in the two groups. A sequence of tests using controlled natural stimuli were conducted at 30 min intervals. Using this stimulation protocol, and controlled regular anesthetic administration (see Methods), the responses were stable over the time of the recording. All of the neurons, in both groups, showed responses to the noxious cutaneous stimulus used (calibrated pinch).

**Table 1:** Average recording depth, antidromic conduction velocity and antidromic stimulation threshold for Lamina I spinoparabrachial neurons recorded from naïve and injured rats.

	*Depth (μm)*	*Conduction velocity (m/s)*	*Threshold (μA)*
Naïve (n = 38)	262 ± 9	9.4 ± 0.6	347 ± 45
Nerve injured (n = 12)	267 ± 13	9.6 ± 0.9	332 ± 47

### Changes in properties and sensory specificity of neurons after nerve injury

#### Responses to noxious stimulation

**Figure 2:**
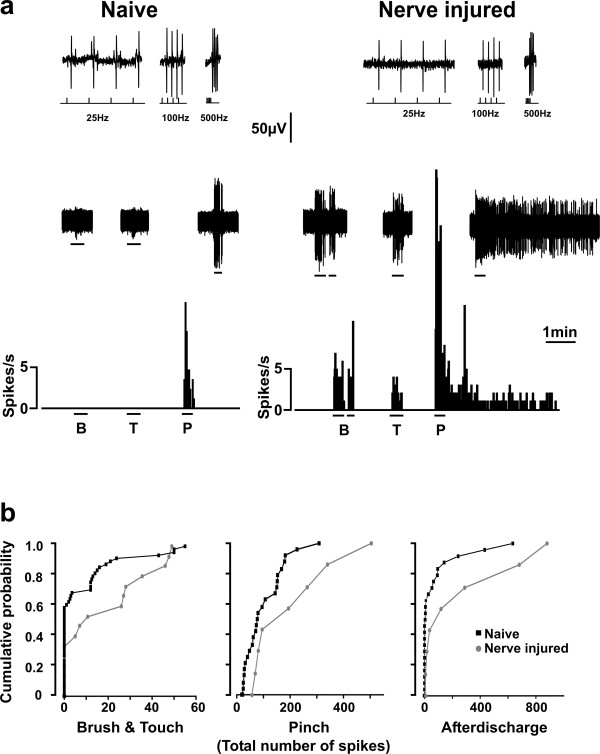
**Nerve injury alters the sensory specificity of lamina I projection neurons**. **a**. The majority (79%) of lamina I projection neurons in naïve rats were nociceptive specific whereas the majority (58%) of those recorded in nerve injured rats responded to both noxious and innocuous stimuli. The rate meter records show representative responses to natural stimuli (B = Brush; T = Touch; P = Pinch) of two identified lamina I projection neurons in a naïve rat and a rat with nerve injury. The inset shows the responses of the cells to trains of stimuli delivered in the parabrachial nucleus at decreasing interspike intervals; the cells followed > 500 Hz stimulation confirming antidromic activation (see methods and Fig. 1b). **b**. The cumulative probability plots show a significant increase in the response to innocuous (data from responses to brush and touch were pooled) and noxious (pinch) stimulation of the hind paw in lumbar spinal lamina I projection neurons after nerve injury. Results are expressed as total number of spikes during the stimulus for response to Brush/Touch and Pinch and throughout the duration of the afterdischarge (in other words, area under the curve).

In control animals, the mean response to calibrated pinch stimuli was 10.1 ± 1.5 spikes/s during the stimulus (Fig. 2). In 38% of these neurons, an afterdischarge was observed after the end of the noxious stimulus (mean duration of 33 ± 11s; Fig. 2). In rats with nerve injury, the mean response to pinch was 22.1 ± 6.1 spikes/s (220% that of control) and was followed in all of the neurons by an afterdischarge (on average, 5 times more prolonged in duration, 160.6 ± 93.4s; p < 0.01; Fig. 2). Thus, lamina I output neurons displayed enhanced nociceptive responses after nerve injury, consistent with hyperalgesia.

#### Responses to innocuous stimulation

In control rats, only 21% (8/38) of the neurons showed any response to either brush or touch stimulation, consistent with previous reports [12]. By contrast, significantly more neurons, 58% (7/12), responded to innocuous input in animals with nerve injury (p < 0.05; Fig. 2). In 34 neurons, the stimuli were repeated a sufficient number of times to allow for a thorough quantification of the responses: the mean response to innocuous stimulation was 270% of control in nerve-injured animals (1.0 ± 0.3 spikes/s; n = 8) as compared with control animals (0.38 ± 0.1 spikes/s; p < 0.05; n = 26; Fig. 2b). Thus, the lamina I nociceptive output pathway incorporated a significant innocuous relay component after nerve injury, consistent with allodynia.

#### Ongoing activity

**Figure 3:**
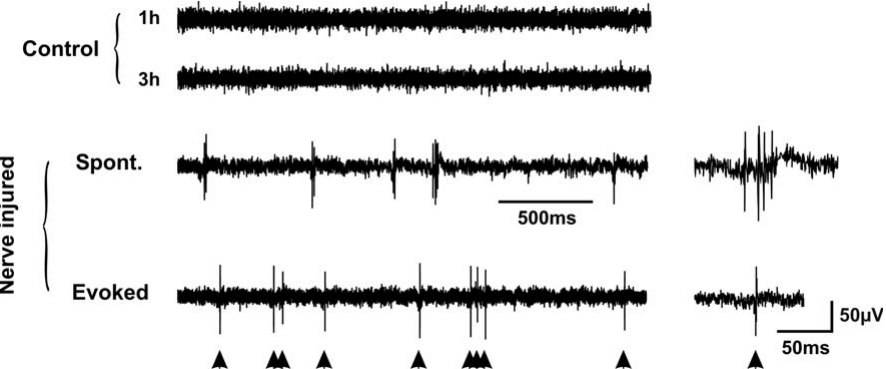
**Occurrence of spontaneous bursts of activity in lamina I projection neurons following nerve injury**. Continuous records showing an example of an epoch with spontaneous bursts of activity occurring in lamina I projection neurons after nerve injury (6 out of 8 where ongoing activity was measured over a sufficiently long period to obtain a quantitative measure). The bursts were characterized by spikes arising on top of a slower wave, which contrasted with the evoked activity in response to touch (lower trace; arrowheads indicated touch stimuli). Such bursts were virtually absent in control animals even after several hours of recording. The insets on the right show examples of spontaneous and evoked activity on a faster time scale.

To address the issue of spontaneous pain, we then examined the ongoing activity of the cells between the periods of overt cutaneous stimulation. In control animals, lamina I projection neurons were virtually silent, in the absence of overt cutaneous stimulation (mean frequency of ongoing firing 0.05 ± 0.02 Hz). In neurons recorded from nerve injured rats, the mean spontaneous firing activity was still very low but significantly increased (0.09 ± 0.01 Hz; p < 0.05). However, the firing pattern of the spontaneous activity was different in the two conditions: in neurons in nerve-injured animals we observed the appearance of spontaneous bursts of activity in absence of any peripheral stimuli which were never observed in control animals (Fig. 3). These bursts were characterized by the occurrence of spikes on top of a slow wave and thus qualitatively different from evoked response to tactile stimuli (Fig. 3) suggesting that they were not evoked by activation of the cutaneous receptive field of the neurons. These spontaneous bursts of spikes in lamina I output neurons may represent a substrate of spontaneous pain that occurs in neuropathic conditions [2,19].

### Effect of acute local spinal administration of ATP-stimulated microglia

Tactile allodynia has been shown to be caused over several hours in naïve animals by local spinal administration of ATP-stimulated microglia [8,20]. We therefore next determined whether acutely administering these microglia centrally, at the spinal level in control rats, could replicate the changes in properties of lamina I projection neurons observed in nerve-injured animals.

#### Responses to noxious stimulation

**Figure 4:**
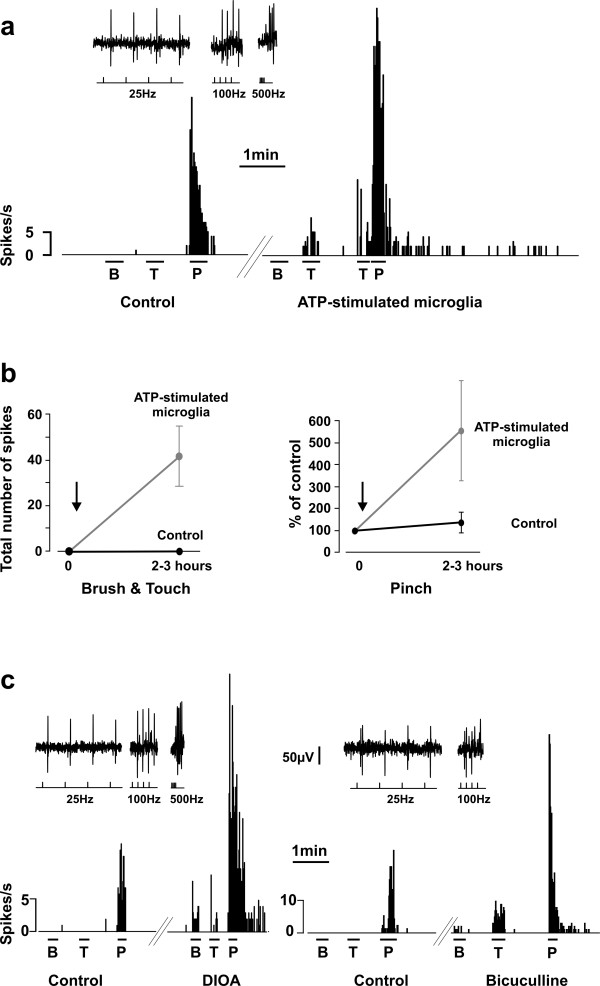
**Stimulated microglia, disruption of chloride homeostasis and bicuculline alter the sensory selectivity of nociceptive specific lamina I projection neurons**. **a**. Rate meter records at the top show the response of an identified lamina I projection neuron to natural mechanical stimulation of the receptive field (B = Brush; T = Touch; P = Pinch). The inset shows antidromic spikes from the parabrachial nucleus following our protocol for identification of projection neurons (see methods and Fig. 1b). **b**. Graphs showing the population data (values indicate mean ± SEM). To avoid biasing the results because of heterogeneity in responses between cells, values are expressed as a percent of control response for each cell. While none of 30 nociceptive specific lamina I projection neuron tested showed occurrence of responses to innocuous input (neither brush or touch) in control conditions after up to 4 hours of recording, all four nociceptive specific cells tested showed a significant response to innocuous input (brush and touch) within 2–3 h of local administration of ATP-stimulated microglia on the surface of the lumbar spinal cord. **c**. Blockade of cation-chloride co-transporters with local spinal administration of DIOA, or blockade of GABA_A _receptors with bicuculline unmasked innocuous input (brush and touch) to nociceptive specific lamina I projection neurons in control animals.

We found that local spinal administration of ATP-stimulated microglia caused a 2 fold increase in mean peak response (21.0 ± 4.8 *vs*. 9.8 ± 3.4 spikes/s; p < 0.01) and a 12 fold increase in afterdischarge duration (192.1 ± 68.0 *vs*. 16.2 ± 8.9 spikes/s; p < 0.05) to calibrated noxious pinch stimulation of the receptive field of lamina I projection neurons (Fig. 4a). To avoid biasing the results, because of heterogeneity in responses between cells, we also express the change in response normalised to the response in control condition for each cell. Using this approach revealed a 5 fold increase in both mean peak response to pinch and afterdischarge duration (p < 0.05; Fig. 4b).

#### Responses to innocuous stimulation

More importantly, in neurons with nociceptive-only responses (n = 4), local spinal administration of ATP-stimulated microglia led to the gradual appearance of responses to brush or touch stimulation, responses which developed over 2 to 3 hours post administration (Fig. 4b). By contrast, in control animals not receiving microglia, neurons with responses only to nociceptive stimulation never developed responses to innocuous stimuli (n = 8 neurons). Thus, ATP-stimulated microglia acutely and dramatically altered the output characteristics of the nociceptive-specific neurons, allowing them to be driven by innocuous cutaneous inputs. Moreover, in cells which showed responses to innocuous input at the onset of recording, administering ATP-stimulated microglia significantly increased the magnitude of the responses to innocuous input (2.8 ± 0.7 *vs*. 1.3 ± 0.4 spikes/s; not shown; p < 0.05; when expressing the values as a percent increase from the response in control conditions for each cell, the ratio was 295 ± 72%).

#### Ongoing activity

**Figure 5:**
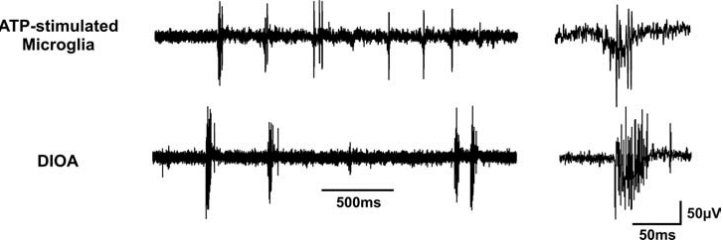
**Stimulated microglia or disruption of chloride homeostasis provoke the occurrence of spontaneous bursts of activity in lamina I projection neurons**. Continuous records showing the appearance of spontaneous bursts of activity in lamina I projection neurons after local administration of ATP-stimulated microglia or DIOA. The bursts were comparable to those observed in lamina I neurons from nerve-injured rats (see Fig. 3). Such burst were virtually absent in control conditions even after several hours of recording. The insets on the right show example bursts on a faster time scale.

Finally, bursts of spontaneous activity were observed after local spinal administration of ATP-stimulated microglia (Fig. 5). Thus, local spinal administration of ATP-stimulated microglia in naïve animals caused a change in the pattern of ongoing discharge of lamina I projection neurons similar to that caused by peripheral nerve injury. Importantly, this effect appears in a time period that is comparable to that within which mechanical threshold significantly decreased following intrathecal administration of ATP-stimulated microglia [8,20].

### Effect of disruption of chloride homeostasis

As ATP-stimulated microglia and nerve injury have previously been shown to cause a collapse in the anion gradient in dorsal horn neurons recorded in vitro [8], we then investigated the effects of disrupting the anion gradient by interfering with ion transport directly.

#### Responses to noxious stimulation

We found that within 1 hour of applying DIOA (100 μM), a blocker of cation-chloride co-transporters [7], responses to noxious stimuli were significantly increased (16.2 ± 3.0 *vs*. 8.5 ± 1.3 spikes/s during pinch stimulation; 63.0 ± 22.3 *vs*. 9.9 ± 6.3 s in afterdischarge duration; n = 7; p < 0.05). When normalizing the response to their control for each cell, the increase in peak response to pinch was 250 ± 32% of control and the increase in afterdischarge duration was 508 ± 110% of control; Fig. 4c).

#### Responses to innocuous stimulation

As with ATP-stimulated microglia, responses to innocuous input were unmasked in previously nociceptive-only neurons (n = 2). Cells with responses to innocuous input showed a significant increase in responses to innocuous input (brush and touch; 2.4 ± 1.2 *vs *1.0 ± 0.6 spikes/s; p < 0.05; when normalizing to control for each cell, the increase in response was 212 ± 40% of control; Fig. 4c). Given our previous finding that impairing chloride homeostasis significantly attenuates GABA_A_/glycine receptor-mediated inhibition [7,9], in an additional cell, we confirmed that local spinal administration of bicuculline (50 μM) could similarly unmask innocuous input to lamina I neurons (consistent with behavioural findings [21-23]). The response to pinch rose from 5.9 ± 1.0 to 15.3 ± 1.3 spikes/s (p < 0.05). Furthermore, while there was no response to innocuous input in control conditions, after bicuculline the average value of the responses to innocuous stimuli was 3.3 ± 0.1 spikes/s (Fig. 4c).

#### Ongoing activity

Finally, bursts of spontaneous activity were observed after local spinal administration of DIOA (Fig. 5). Therefore, disruption of chloride homeostasis in the superficial dorsal horn is sufficient to replicate the effect of nerve injury on spinal lamina I output.

## Discussion

Our results show that in control conditions less than 25% of mechanosensitive lamina I projection neurons produce action potentials in response to low-threshold tactile stimuli, consistent with previous studies in rats [12] and cats [14,24]. In contrast to the situation in naïve animals we show here for the first time that peripheral nerve injury causes a functional switch in the sensory specificity of this subpopulation of spinal output neurons whereby the majority of these neurons respond to low-threshold tactile stimuli. Finally, the same qualitative functional switch is triggered in naïve animals by acute local, spinal, application of ATP-stimulated microglia or disruption of chloride homeostasis.

Hyperalgesia can be explained by a quantitative change in response properties of nociceptive relay neurons whereby the same nociceptive input generates a greater action potential output. However, given that the majority of lamina I neurons respond to noxious input only, a quantitative change in nociceptive responsiveness appears insufficient to explain allodynia. Rather, allodynia implies a qualitative change, a miscoding of information such that innocuous inputs are converted into a nociceptive message. The switch in modality specificity we observed in lamina I output neurons is such a qualitative change in response properties and thus may be sufficient to explain allodynia. After peripheral nerve injury, administering ATP-stimulated microglia or disrupting chloride transport, innocuous inputs are transformed in the dorsal horn and become encoded by lamina I projection neurons. Consequently, action potential discharge is now generated by these neurons and sent to higher brain structures through output neurons that were previously nociceptive specific. It is thus logical to infer that such signals will be interpreted as noxious at the supraspinal level, providing a substrate to explain tactile allodynia. Similar logic can be applied to the finding of the appearance of spontaneous bursts of spikes in lamina I output neurons after nerve injury, treatment with ATP-stimulated microglia, or disruption of chloride homeostasis, providing a substrate of spontaneous pain that occur in neuropathic conditions.

A comparable loss of selectivity for noxious stimuli has been observed on unidentified nociceptive specific neurons in the superficial dorsal horn following application of mustard oil or capsaicin on the receptive field of the cells in the periphery [25,26]. Similarly, brushing of the skin after sciatic nerve crush leads to c-fos expression in the superficial dorsal horn [27]. Under those conditions, however, as with nerve injury, the possibility remains that the central response to low-threshold stimuli is due to a peripheral change in selectivity (e.g. peripheral nociceptors responding to low-threshold mechanical stimuli). In contrast, the evidence presented here, using local spinal administration of agents, shows conclusively that responses to low threshold inputs by lamina I neurons can result from purely central mechanisms.

Based on previous studies, the proposed central mechanism that was affected in the current experimental conditions is a disruption of anion homeostasis [7,8], effectively weakening inhibition [9]. The site at which the disinhibition occurs appears to be within the circuitry intrinsic to the dorsal horn, not within the network of afferent or descending terminals entering the dorsal horn. A loss of KCC2, which normally extrudes Cl^- ^from the cells, appears to be the underlying mechanism [7,8,28] and KCC2 is not present in synaptic terminals nor on primary afferents [7]. The other principal regulator of Cl^- ^in the dorsal horn is NKCC1, which normally causes Cl^- ^accumulation into cells. NKCC1 is very weakly expressed in adult dorsal horn neurons [29,30] but is the dominant cation-chloride co-transporter in primary afferents [31], and thus NKCC1 leads to GABA exerting a depolarizing, albeit inhibitory, action on sensory terminals. Abnormal presynaptic excitation of sensory terminals has been proposed to occur via upregulation of NKCC1 in small diameter afferents after an inflammatory peripheral insult [31]. This is thought to produce suprathreshold GABAergic depolarization in sensory terminals yielding cross excitation between low and high threshold afferents. The latter mechanism is unlikely to contribute to the effects observed in the present study for the following reasons. First, nerve injury is associated with a loss of KCC2 expression [7] as mentioned above. Second, ATP-stimulated microglia has been shown to cause tactile allodynia via the release of BDNF [8], and BDNF-trkB signalling is linked to downregulation of KCC2 [28,32]. Third, the blocker of cation-chloride co-transport used in the present study, DIOA, preferentially inhibits KCC2 and not NKCC1 [33,34]. Even if one doubts the specificity of DIOA and assumes that it also antagonized NKCC1 [35], this site of action could not account for the effect of DIOA we observed, unmasking low-threshold input to lamina I neurons, because blocking NKCC1 would work against exaggerated depolarization in primary afferents and thus prevent rather than produce cross talk between them as proposed for inflammatory insult [36]. In summary, our findings indicate that selective impairment of postsynaptic Cl^- ^homeostasis in the spinal dorsal horn is sufficient to unmask the relay of innocuous input through normally nociceptive specific pathways.

This aberrant relay of innocuous input may occur via unmasking polysynaptic connections in the superficial dorsal horn [37-39] functionally linking low threshold afferents and nociceptive lamina I projection neurons [6]. This can be mediated either via disinhibition of feedforward excitatory interneurons such that they can convey this input onto normally nociceptive lamina I neurons or by lowering the threshold of nociceptive lamina I neurons to normally subthreshold polysynaptic input [40]. In addition, unmasking of low-threshold input to lamina I output neurons may occur via inversion of normally inhibitory post-synaptic events from inhibitory interneurons into excitatory ones in a subset of cells [7].

The finding that altered chloride homeostasis compromises inhibitory control in dorsal horn neurons raises the question of the therapeutic avenues to compensate for this form of disinhibition. Indeed, a weakening of the hyperpolarizing action of GABA_A_/glycine receptor activation suggests that drugs aimed at enhancing GABA_A_/glycine receptor-mediated inhibition may be ineffective in reversing nerve injury-induced allodynia. However, three elements must be considered before making such inference. First, while raising intracellular [Cl^-^] suppresses the component of inhibition caused by hyperpolarizing the neuron, it only minimally affects the component of inhibition caused by shunting the membrane (as discussed in detail in [9]). Second, it is particularly important to note that a small depolarizing shift in reversal potential for GABA_A _currents will cause a loss of inhibition without necessarily causing GABA_A_-mediated net excitation. Thus, when intracellular Cl^- ^homeostasis is altered, activation of GABA_A _and glycine receptors may continue to be inhibitory albeit less inhibitory [9,10], allowing for unmasking of latent excitatory inputs. Finally, it must be kept in mind that because presynaptic GABA_A _receptor-mediated inhibition remains intact (see above), drugs activating or enhancing the function of GABA_A _receptors may remain analgesic by inhibiting afferent input at its entry point into the spinal cord. This is consistent with reports of antiallodynic effect of intrathecally-applied GABA_A _receptor agonists [41,42]. Thus, at least in some cases, sufficient residual inhibition may remain in neuropathic pain conditions permitting GABAergic drugs to be analgesic. Results from modelling studies show that while small reductions in anion gradient may be effectively compensated for by potentiating GABA_A_/glycine receptor-mediated input, this can occur at the expense of stability of the system, and compensation will fail as the reduction of anion gradient exceeds a critical value [9]. Depending on the extent of the pathology, compensation by enhancing GABAergic transmission may therefore be effective or not. Thus, measuring intracellular [Cl^-^] may be important to guide treatments based on GABA-modulating agents, and restoring normal anion homeostasis or targeting excitatory transmission may represent more effective therapeutic strategies [10].

## Conclusion

The changes in response properties, selectivity and discharge activity of lamina I output neurons caused by ATP-stimulated microglia or by disrupting chloride homeostasis reproduce the changes observed after peripheral nerve injury. These changes appear to match the cardinal symptoms of neuropathic pain and are thus sufficient to explain at least some forms of the pathology, providing novel avenues for the treatment of this highly debilitating condition.

## Methods

All experimental procedures were performed in accordance with guidelines from the Canadian Council on Animal Care.

### Animal Preparation

Male Sprague-Dawley rats (post-natal day 60+; 300–350 g, Charles River Laboratories, Wilmington, MA) were anaesthetized by intraperitoneal injection of pentobarbital (initial dose: 0.65 mg/kg; subsequent doses 0.35 mg/kg every hour). To maintain a stable level of anaesthesia, rats were given a supplemental dose of anaesthetic every hour post surgery. To test for possible influence of the anaesthesia on our recordings, Ketamine/Xylazine (0.1 ml/100 g) was used in some experiments. No difference was observed under either condition (data not shown).

Spinal cord segments L4-S1 were exposed by laminectomy. The jugular vein was canulated and a tracheotomy performed. The rat was then mounted on the stereotaxic frame and the vertebrae stabilised using two spinal clamps. A small recording chamber was made around the exposed spinal cord segment using agar (3%) to isolate the exposed spinal cord and prevent the diffusion of the liquid outside this area.

Animals were administered pancuronium bromide (Sigma, 5 mg/ml; 0.1 ml initially and then 0.05 ml hourly) and ventilated artificially. Expired CO_2 _was maintained at 4%. The body temperature was continuously controlled and maintained at 37.5°C. Immediately before recording, the meninges were carefully removed and the spinal cord covered with mineral oil at 34°C to avoid drying of the spinal cord.

### Peripheral nerve injury

Peripheral nerve injury (PNI) was performed by surgically implanting a polyethylene cuff around the sciatic nerve of anaesthetized adult rats as described previously [7,43,44]. Paw withdrawal threshold was measured using von Frey filaments to demonstrate tactile allodynia as described previously [7,45]. Within the nerve injured group, only animals that showed a gradual decrease in mechanical threshold (over 14–17 days) down to 2.0 g or less were used for recordings.

### Recordings and stimulation

Extracellular single unit recordings were conducted using stainless steel metal electrodes (10 MΩ, FHC, USA; ER-1 extracellular amplifier, Cygnus technology). The recording electrode was mounted on a high precision manipulator (Burleigh 6000 controller) and the zero was set as the electrode touched spinal cord surface. The signal was filtered between 300 Hz and 1.5 kHz (Brownlee precision).

To identify lamina I projection neurons, the lateral parabrachial nucleus was stimulated as described previously [12]. An array of 6 electrodes was specially designed to fit within the parabrachial nucleus (staggered by 200 μm; contact diameter 100 μm; contact length 150 μm; custom-made by Peter Rhodes, Rhodes Medical Instruments Inc). Stimulation of the parabrachial nucleus consisted of a train of 4 stimuli. Units were confirmed as projection neuron if they followed the train of 4 antidromic stimuli delivered at up to 500 Hz or if collision between antidromic and orthodromic action potentials was observed (Fig. 1b). Importantly, only one lamina I projection neuron per rat was recorded as each neuron was taken as its own control during treatments with either drug or microglia.

We used an established routine to characterize the response of lamina I projection neurons to innocuous and noxious natural stimulation of the receptive field: 20s of brush, 20s of repetitive touch (with the tip of finger, yielding 80 touch/20s) and 10s of noxious pinch using calibrated forceps (100 g; 1 mm diameter tip; Electrotechnology Selem Inc, Québec, Qc, Canada). Each set of stimuli was delivered at 30 min interval. Detailed mapping of the receptive fields as well as potential changes in receptive field size was not conducted to avoid applying repeating stimuli too often, which has been shown to produce sensitization of lamina I cells [14].

### Data acquisition and analysis

Extracellular recordings, parabrachial stimulation and expired CO_2 _were sampled at 20 kHz and stored on a computer using an analog-to-digital conversion system (Powerlab 8SP, AD instruments Inc.). Analysis was performed offline using Neuroexplorer (Nextechnology, USA) and locally designed software. Mean response to sensory stimulation (i.e. cumulative number of spikes detected during the stimulation period or mean frequency) was calculated for each neuron before and after drug treatment.

### Microglia cultures

Rat primary cultures were prepared from neonatal cortex or spinal cord as previously described [6,7] and maintained for 10–14 days in DMEM medium with 10% fetal bovine serum. Microglia were separated from the primary culture by gentle shaking of the flask and were replated on plastic dishes. The cells were removed from the dish surface with a cell scraper and collected in 100 ml of PBS buffer. This method produces microglia cultures of >98% purity. The density of microglia was measured using a haemocytometer and the volume of PBS was adjusted to give a final density of 1,000 cells per 10 μl. Microglia were stimulated by incubation with 50 μM ATP for 1 hour.

### Drugs

Drugs were applied directly on the top of the spinal dorsal horn using the chamber described above. R(+)DIOA (Sigma, 100 μM) was first prepared in ethanol at 100× concentration before being diluted in 0.9% NaCl. Bicuculline HCl (Tocris, 50 μM) was dissolved in saline 0.9%NaCl. Microglia were suspended in PBS. All solutions were applied at 34°C.

### Statistics

All results are expressed as mean ± SEM. An arcsine transformation was performed to correct for binomial distributions when data were expressed as percentages or proportions [46]. Chi squared test for contingency tables were used to compare proportion of lamina I projection neurons with and without responses to innocuous stimuli in control rats before and after drug application and in rats with nerve injury. Nonparametric Mann-Whitney post-hoc test was used to compare unpaired data whereas Wilcoxon post-hoc paired test was used to assess the effect of drug applications. Statistical significance was set at p < 0.05.

## Competing interests

The author(s) declare that they have no competing interests.
